# Genomic Analysis of *Corynebacterium diphtheriae* Strains Isolated in the Years 2007–2022 with a Report on the Identification of the First Non-Toxigenic *tox* Gene-Bearing Strain in Poland

**DOI:** 10.3390/ijms24054612

**Published:** 2023-02-27

**Authors:** Tomasz Wołkowicz, Katarzyna Zacharczuk, Aleksandra Anna Zasada

**Affiliations:** 1 Department of Bacteriology and Biocontamination Control, National Institute of Public Health NIH—National Research Institute, 24 Chocimska Str., 00-791 Warsaw, Poland; 2 Department of Sera and Vaccines Evaluation, National Institute of Public Health NIH—National Research Institute, 24 Chocimska Str., 00-791 Warsaw, Poland

**Keywords:** *Corynebacterium diphtheriae*, diphtheria toxin, whole genome sequencing, NTTB strains, MLST typing, non-toxigenic

## Abstract

Infections caused by non-toxigenic *Corynebacterium diphtheriae* have been reported every year in Poland since 2004, with the ST8 biovar gravis strains being most commonly isolated. This study analyzed thirty strains isolated between 2017 and 2022 and six previously isolated strains. All the strains were characterized using classic methods in terms of species, biovar level, and diphtheria toxin production, as well as by means of whole genome sequencing. The phylogenetic relationship based on SNP analysis was determined. The number of *C. diphtheriae* infections has been rising in Poland every year with a maximum of 22 cases in the year 2019. Since 2022, only the non-toxigenic gravis ST8 (most common) and mitis ST439 (less common) strains have been isolated. An analysis of the genomes of the ST8 strains showed that they had many potential virulence factors, such as adhesins and iron-uptake systems. The situation rapidly changed in 2022 and strains from different STs were isolated (ST32, 40, and 819). The ST40 biovar mitis strain was found to be non-toxigenic *tox* gene-bearing (NTTB), with the *tox* gene inactivated due to a single nucleotide deletion. Such strains were previously isolated in Belarus. The sudden appearance of new *C. diphtheriae* strains with different STs and the isolation of the first NTTB strain in Poland indicate that *C. diphtheriae* should be classified as a pathogen of special public health concern.

## 1. Introduction

*Corynebacterium diphtheriae* constitutes an etiologic agent of diphtheria, a life-threatening disease involving local infections of the respiratory tract and other mucus membranes, complicated by the effects of produced diphtheria toxin that causes early damage to the heart muscle fibers, nerve demyelination, and necrosis. Notably, diphtheria can also be caused by the *C. ulcerans* and *C. pseudotuberculosis* strains, but it is less common. Diphtheria was a serious public health problem in the past, but it has largely been brought under control thanks to the introduction of compulsory vaccination against the disease in the 1940s. However, the vaccine used globally contains only diphtheria toxoid and does not prevent infection caused by non-toxigenic strains [1]. Diphtheria toxin is the most important virulence factor of the *C. diphtheriae* strains and, because of that, non-toxigenic strains have been regarded as non-pathogenic. In most cases, such strains lack the *tox* gene that encodes the toxin. A specific group of non-toxigenic strains is made up of non-toxigenic *tox* gene-bearing (NTTB) strains that have the *tox* gene in their genome, although inactivated by different mechanisms [2].

An increasing number of cases of invasive infections caused by non-toxigenic isolates has recently been reported in many countries with high vaccination coverage [3,4,5]. Such invasive infections have a high mortality rate [6]. Several risk factors of invasive non-toxigenic *C. diphtheriae* infections have been identified—mainly homelessness, alcohol abuse, diabetes mellitus, cardiac diseases, intravenous drug use, hepatic cirrhosis, and dental caries [1,7]. In Poland, the first observed case of an invasive infection caused by non-toxigenic *C. diphtheriae* was described in 2004 [8]. Since that time, such infections have been recorded every year. Previously conducted studies revealed that all the non-toxigenic *C. diphtheriae* strains isolated from invasive and local infections in Poland in the years 2004–2013 belonged to the same biotype, gravis, all shared the same ST-8 type in MLST, and were undistinguishable by other genotyping methods such as PFGE, ribotyping, MLVA, and ERIC-PCR [4,6]. It was found that previously, the ST8 strains belonged to the clonal complex associated with the recent big diphtheria epidemic in the former Soviet Union in the 1990s, which affected the whole of Europe. However, the epidemic strain carried the *tox* gene, while all the mentioned strains isolated in Poland were non-toxigenic. Despite the fact that Poland was geographically close to the Soviet Union, the country was protected from the diphtheria epidemic by high immunization coverage [9]. It is possible that the loss of the *tox* gene enabled the ST8 strains to survive among the highly vaccinated Polish population and cause serious invasive infections. The diphtheria toxin gene is located in the genetic material of bacteriophage β and only lysogenized strains are able to produce the toxin, but the toxin production is regulated by chromosomal regulatory genes, especially the iron-dependent *dtxR* repressor [10].

The aim of this study was to analyze the *C. diphtheriae* strains isolated from human infections in Poland in recent years, mostly 2017–2022. To date, there are no whole genome sequencing data and analyses available from Poland in this field.

## 2. Results

**Figure 1:**
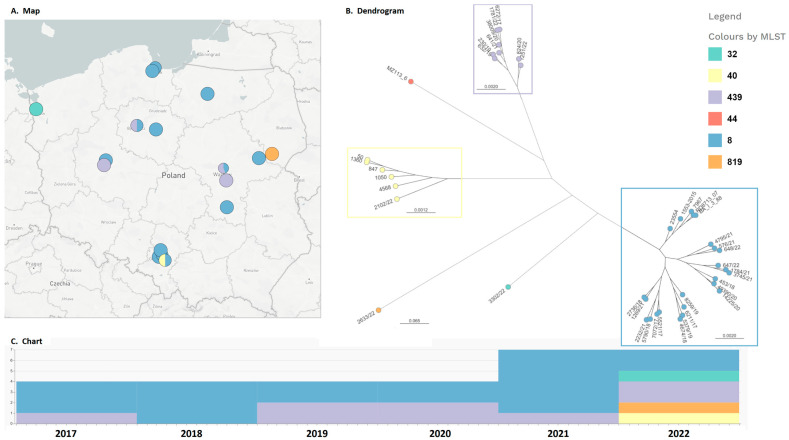
The geographic, time-related, and phylogenetic distribution of the analyzed strains. The whole Microreact analysis is available online (a link to the relevant project is provided in the text).

In Poland, like other European countries, only diphtheria (toxin-positive) cases are reported to the national epidemiological system, which is why there are no large pools of epidemiological data relating to analyzed cases. All the primary data relating to the analyzed strains are shown in Table 1. An analysis of basic clinical data shows that, among 30 tested strains isolated between 2017 and 2022, 13 (43%) were isolated from blood, 9 (30%) were isolated from wounds, 4 from respiratory tracts, and one from an eye. In three cases, there is a lack of information about the source or site of infection. In five cases, the strains were isolated from homeless people, while in another five cases, there was no address information on the diagnostics study commission. The patients’ ages ranged from 17 to 90 years old with the median at 53.5. The geographical distribution of the analyzed strains with all the basic data is shown in Figure 1 and in the Microreact project available online (https://microreact.org/project/dWfYQoVtmA6Rqah76tN3KS-corynebacterium-in-poland-2017-2022-with-background, accessed on 13 December 2022) [11].

A phenotypic analysis shows that 20 strains isolated since 2017 belonged to the *C. diphtheriae* gravis biotype. Additionally, five previously isolated strains (years 2007–2015) also belonged to the *C. diphtheriae* gravis biovar. Only 10 belonged to the *C. diphtheriae* mitis biovar. Four of these mitis strains were isolated this year (only until August). For all the 30 strains from the years 2017–2022, sequenced on GridION, it was possible to assemble the genomes into one complete and circular chromosome, without any plasmids in most strains. All the genomes have a similar size, with an average of 2,444,112 bp. One mitis strain has a bigger genome (2,506,552 bp), while another mitis strain has a smaller genome (2,362,113 bp). Both of these outlier strains were isolated in 2022.

All 25 *C. diphtheriae* gravis biovar strains were negative in terms of diphtheria toxin, both in the ELEK test and in PCR. The *sul1* gene was present in 14 strains and it was the only resistance gene found in these strains. A classic seven-gene MLST assay determined that twenty-four strains (96%) belonged to ST8 and one belonged to ST32. An analysis of genes coding different virulence factors showed that the analyzed gravis ST8 strains were better equipped than the mitis strains and had all the verified genes present in their genome (except the diphtheria toxin gene, even when searching with low similarity and length thresholds). The *C. diphtheriae* biovar gravis ST32 strain had neither the DIP0543 (neuraminidase) nor DIP2093 (putative fimbrial adhesin) genes, nor the whole SpaD-type pili gene cluster. All the results are shown in Table 2.

Moreover, all the tested mitis strains were negative in terms of diphtheria toxin in an ELEK test, but one strain isolated in 2022 was positive in PCR tests. Eight strains belonged to ST439, one belonged to ST819, and the NTTB strain belonged to ST40. No antimicrobial resistance determinants were found in these strains. In general, the mitis strains had fewer virulence factor determinants with the lack of the SpaH-type pili cluster, DIP2093 (putative fimbrial adhesin), and DIP0543 (neuraminidase). In the mitis strains, the SpaA-type and the SpaD-type pili clusters are slightly different (around 85–96% of sequence identity). Additionally, strains from ST439 lack the *spaB* and *spaD* genes. In comparison, the whole SpaD-type pili gene cluster and the *spaC* gene (part of the SpaA-type pili cluster) were not detected in the other analyzed mitis STs.

More detailed analyses were performed on the sequence of the *tox* gene of the *C. diphtheriae* ST40 strain. It was found that the diphtheria toxin gene was complete, but a single nucleotide deletion at position 55 (deletion of a single guanidine) resulted in an early frameshift, and a disruption of the gene was found. The same single-nucleotide deletion was found when compared to all the strains isolated previously in Belarus. An attempt was made to estimate the frequency of the occurrence of this mutation by searching for it using the BLASTn software, and only four more of such sequences deposited in the GenBank database (strains isolated in the UK, Belgium, Russia, and Australia, according to data in the GenBank) were found.

A complete dendrogram based on the SNP analysis is shown in Figure 1. A phylogenetic analysis confirmed that all the gravis strains that belonged to ST8 were closely related. The five oldest strains can be distinguished as a separate, closely related subgroup. This should be analyzed carefully because of the fact that these strains were sequenced only using the Illumina platform and, as a result, a different assembling algorithm. Another subcluster can be distinguished with strains 576/21, 1784/21, 3745/21, 4795/21, 647/22, and 648/22. These strains were isolated in the years 2021–2022 and, except for the oldest strain that was isolated in Warsaw, all the other strains were isolated in the Silesia Voivodeship. Moreover, strains 453/18, 49390/20, and 14225/20 can be distinguished as a separate subcluster, but in this case, all these strains were isolated from different Polish regions. The last subcluster of the gravis strains can be distinguished with strains 5521/17, 7072/17, 5790/18, 2736/18, 1269/21, and 2232/21, but these strains were isolated for an extended period of time (2017–2021) and across a huge area of central-northeastern Polish regions.

Strains from the mitis biotype ST 439 can constitute a separate, internally quite consistent group, with only strains 824/20 and 1251/22 that can be distinguished as a subcluster. Although the NTTB mitis strains belonging to ST40 are grouped as a completely separate cluster, strain 2102/2022 isolated in Poland in 2022 is relatively different (172 to 217 SNPs found in comparison to 23–96 SNPs between other strains). These strains were obviously isolated in different periods of time, which can explain such a number of SNPs observed.

All the whole genome sequences were deposited in the GenBank public database as part of BioProject No. PRJNA873913.

## 3. Discussion

**Figure 2:**
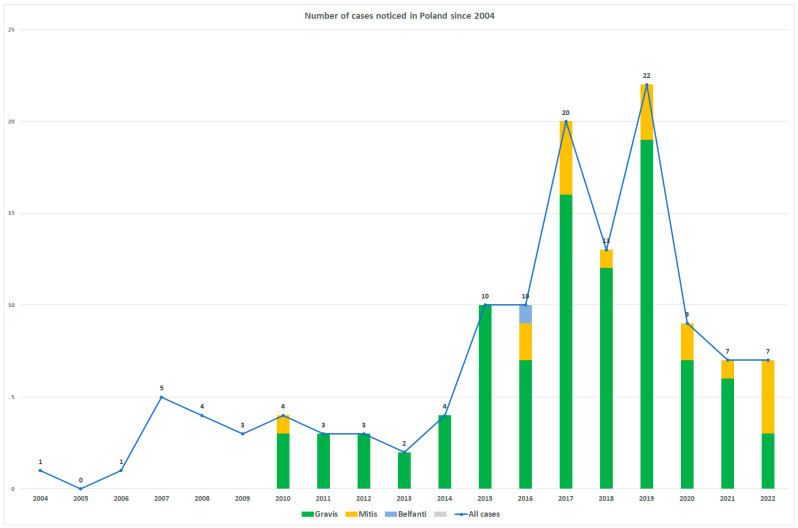
The number of *C. diphtheriae* infections recorded in Poland since 2004.

The last diphtheria case in Poland was recorded in 2000, and since 2004, only infections caused by non-toxigenic *C. diphtheriae* have been observed every year (data from the Department of Bacteriology and Biocontamination Control of National Institute of Public Health NIH–NRI). As shown in Figure 2, before the COVID-19 pandemic, the number of such infections increased over the years, with a maximum of 22 cases in 2019. These data confirm that non-toxigenic *C. diphtheriae* should be treated as a re-emerging pathogen. During the COVID-19 pandemic, the number of many different infections significantly decreased, and a similar decrease can be observed in the *C. diphtheriae* cases. Determining whether that was because of a lower number of infections or the shift of diagnostic scope towards SARS-CoV-2 detection causing many different infections to remain undetected is obviously difficult.

The non-toxigenic gravis strains from ST8 are predominant in Poland and their recorded number has been stable over the years. Between 2011 and 2015, only *C. diphtheriae* strains from this biovar were isolated in Poland. Since 2016, the mitis biotype strains have also been observed every year but at a much lower number (one to four cases per year), while before 2022, all these mitis strains belonged to ST439. Therefore, the picture of the *C. diphtheriae* strains isolated in Poland before 2022 looks rather monotonous and it was limited to only two biovars and two sequence types. These data correspond to a previous analysis performed by Czajka et al. [12], where the non-toxigenic gravis ST8 strains were also definitely the most frequently observed. This suggests these strains can be endemic to Poland. In contrast, in neighboring Germany, 20 different STs were identified among 76 strains from 2016 to 2017, but the most commonly identified were also ST8 (54%) and ST439 (7%), as well as ST130, which has not been observed in Poland (13%) [3]. Interestingly, ST8 *C. diphtheriae* was not recorded in Germany until 2015. In a comparable period of time, in Austria, around four to eight cases of *Corynebacterium* infections were observed every year (a maximum of thirteen cases in 2014), but the strains were much more diverse (mostly the belfanti, mitis, and gravis biovars) [13]. An additional MLST analysis of the Austrian strains confirmed this high diversity (34 different STs among 57 strains were identified) with no ST8 and only four ST439 strains. An analysis performed on the other side of Poland, in neighboring Belarus, showed that ST5 and ST8 *C. diphtheriae* were most frequently recorded [14]. Furthermore, in this study, ST5 included strains assigned to the belfanti, gravis, and mitis biovars and were non-toxigenic, whereas ST8 strains were toxigenic. Previously, such toxigenic ST8 *C. diphtheriae* strains were often isolated during the epidemic in the 1990s in the former Soviet Union (FSU) and were still isolated both in Belarus and Russia in the post-epidemic period [14,15]. Moreover, Borisova et al. [15] showed that, in Russia, most of the toxigenic strains isolated there in the years 2002–2012 belonged to the gravis biovar and ST8. These data suggest a spread of pathogenic ST8 *C. diphtheriae* from Eastern to Western Europe, correlated with the loss of the *tox* gene and the transformation to a non-toxigenic strain due to the high vaccination rate in Poland and other Western European countries [3,16].

An analysis of different virulence factors showed that the *C. diphtheriae* ST8 strains are better equipped, which may somehow explain their epidemiological success and stable existence in Poland. In these strains, many genes and gene clusters involved in adhesion were found. The SpaA-type pili have been shown to interact with the pharyngeal epithelial cells, while the SpaD-type and SpaH-type pili are responsible for adhesion to the lung and laryngeal epithelial cells [17,18,19]. A complete DIP2093 gene was also found that was shown to encode collagen-binding proteins [20], as well as DIP1621 and DIP1281, which both could play a role in adhesion to epithelial cells [21,22]. These data correspond to the analysis of Belarusian strains and the authors’ hypothesis about their greater abilities to adhere to and invade host cells [14].

On the other hand, such conclusions should be made with caution, as most of such analyses use *C. diphtheria* NCTC 13129 strains as a reference, and this strain belongs to the ST8 gravis biovar and was isolated in Russia during the epidemic in the 1990s. In our analysis, some virulence genes and clusters, such as the SpaA-type pili cluster or SpaD-type pili cluster, had low sequence similarity (around 85–96%) with a lack of some genes in the clusters. Some of these genes were also previously found to be pseudogenes [23]. These differences suggest that some of these virulence determinants have different genetic organization and characteristics within this biovar.

In 2022, the invariable situation in Poland changed and an increased number of infections with strains belonging to other STs and/or the mitis biotype has been reported. Our analysis revealed that, during that year, the first ST40 NTTB, ST32, and ST819 strains appeared. The reason for this sudden variation remains unclear and, because of the lack of proper epidemiological data, can be only speculative. The main hypothesis is associated with the war in Ukraine that began on 24 February 2022 (just before the first different strains were isolated on 17 May) and the related significant migration of Ukrainian refugees to the whole territory of Poland. This hypothesis could especially explain the emergence of the ST40 NTTB strain, which has the eastern link, with strains isolated previously in Belarus.

Unfortunately, there is no clear information on the ST819 strains in the literature, with only one such strain reported in the *Corynebacterium* BigsDb by the Bavarian Health and Food Safety Authority (LGL), which suggests that this ST was previously recorded in Germany. On the contrary, the ST32 gravis strains have previously been widely reported in Europe [13,24,25], but also worldwide, e.g., in Canada [26] and Australia [27]. The ST32 strain described herein was found in a patient in Szczecin, located in the western part of Poland, close to the German border, and it is possible that it was transferred from the west. It was suggested that the spreading success of this clone is due to its superior adherence properties [19]. However, in this study, the SpaD-type pili gene cluster, or RS23695, which seems to encode collagen-binding proteins, was not found [20], but, on the contrary, all these genes were found in ST8 strains.

The NTTB strains require special attention due to the potential possibility of reverting the *tox* gene to the functional version by a spontaneous mutation or homologous recombination between different corynebacteriophages. That is why there has been a special focus on the ST40 NTTB strain isolated in 2022. As previously mentioned, Grosse-Kock et al. [14] found such strains in Belarus in the years 1996–1999 and in 2007. Moreover, Zakikhany et al. [2] found one such strain in the UK, but in this case, it was isolated not from a human, but from a cat. A comparative analysis of the *tox* gene sequence has shown that the Polish ST40 strain has the same single nucleotide deletion in the *tox* gene in position 55 as the strains isolated in Belarus and in the UK. Such point deletions seem to often be the cause of a frameshift and *tox* gene inactivation in the NTTB strains. The same point mutation was found in strains belonging to different STs in Australia [28]. Moreover, a Russian analysis of the NTTB strains isolated in the years 1994–2002 suggests that the deletion of one nucleotide usually inactivates the *tox* gene [29]. There are also different causes of *tox* gene inactivation in the NTTB strains, including mutations in the *dtxR* gene, which encodes the diphtheria toxin regulator, and in the promoter region of the *tox* gene [2,28].

The main limitation of this study is a lack of precise epidemiological data because of the fact that infections caused by non-toxigenic strains are not reported in the national epidemiological systems. As a result, it is, for example, not clear how the NTTB strain ST40 appeared in a Polish citizen from Katowice, which is a city in the Silesia region (geographically located in the south of Poland), far from the Polish eastern border.

In conclusion, the sudden appearance of new *C. diphtheriae* strains with different STs and the isolation of the first NTTB strain in 2022 in Poland indicate that *C. diphtheriae* should be a pathogen of special public health concern, and effective national surveillance is essential.

## 4. Materials and Methods

### 4.1. Bacterial Strains

In this study, 16 randomly selected *C. diphtheriae* strains isolated in Poland in the years 2017–2020 (4 strains from each year) were used. These strains were selected from all the strains sent for routine diagnostics and collected in the Department of Bacteriology and Biocontamination Control of the National Institute of Public Health NIH–NRI in Warsaw, which should represent all strains isolated from clinical samples in Poland. Additionally, 7 strains isolated in 2021 and 7 strains in 2022 (up to August) were sequenced, which constituted all strains collected during these years. Additionally, as a background, 6 previously isolated strains were analyzed. Out of these strains, 5 were isolated between 2007 and 2015 in Poland, while one strain was toxigenic and was isolated from a patient with diphtheria in the 1990s.

### 4.2. Species Verification and Toxin Identification

The strains were identified as *C. diphtheriae* using Gram staining; colonies’ morphology on Columbia agar with 5% sheep blood, Clauberg agar, and Tinstale agar; and a biochemical assay using the API Coryne system (BioMerieux). Diphtheria toxin production was tested using the ELEK test according to the WHO Manual [30]. The occurrence of the *tox* gene-encoding diphtheria toxin was tested with PCR according to Pallen et al. [31] for the active part of the toxin and Hauser et al. [32] for the whole toxin gene.

### 4.3. Whole Genome Sequencing

Whole genome sequencing was performed in different periods of time, separated by the pandemic period. As a result, different sequencing platforms were used for older and newer strains. Sequencing paired-end libraries of the 6 oldest strains were performed using the Illumina Nextera XT kit and were sequenced on the Illumina NextSeq 500 platform. Raw reads were assembled using SpaDES 3.11.0 [33] and CLC Genomics Workbench and merged with CISA [34]. The library preparation and sequencing were performed in the Biobank Lab, University of Łódź.

The sequencing of the 16 strains isolated in the years 2017–2020 was performed using the Illumina and Oxford Nanopore Technologies (ONT) platforms. Illumina paired-end libraries were performed using the Illumina DNA Prep kit, while whole genome sequencing was carried out on the MiSeq instrument. ONT libraries were performed using Rapid Barcoding Kit 96 and sequenced for 26 h on GridION using R9.4 Flow Cells and the Super Accurate basecalling algorithm. Hybrid assembly was performed using the CLC Genomics Workbench and the NanoForms online server [35]. The last 14 strains were sequenced using only the ONT GridION platform, as described previously. Assembly was performed using the CLC Genomics Workbench and the NanoForms online server. For further analysis, sequences assembled with NanoForms were used.

### 4.4. WGS Data Analysis

In the first step of the analysis, species were confirmed using CGE Tools available at http://www.genomicepidemiology.org/ (accessed on 1 September 2022): SpeciesFinder 2.0 [36] and KmerFinder [37]. Multilocus sequence typing (MLST) and the occurrence of AMR (Antimicrobial Resistance) genes were analyzed using MLST 2.0 [38] and ResFinder 4.1 [39], respectively. The hypothetical pathogenic potential was estimated by proteome analysis using PathogenFinder 1.1 [40]. The virulence factor profile was analyzed with MyDbFinder using a FASTA file containing sequences of the following genes: diphtheria toxin gene (*tox*), diphtheria toxin regulator (*dtxR*), SpaA-tyle pili cluster (*spaA, spaB, spaC, srtA*), SpaD-type pili cluster (*spaD, spaE, spaF, srtB, srtC*), SpaH-type pili cluster (*spaG, spaH, spaI, srtD, srtE*), surface-anchored pilus cluster (*sapA, sapD*), ABC transporter cluster (*fagA, fagB, fagC, fagD*), ABC-type haem transporter cluster (*hmuT, hmuU, hmuV*), siderophore-dependent iron uptake system (*irp6A, irp6B, irp6C*), ciu iron uptake and siderophore biosynthesis system cluster (*ciuA, ciuB, ciuC, ciuD, ciuE*), neuraminidase (RS14030), hemagglutinin (RS14950), genes involved in adhesions (RS17590, RS19245, RS23695), and membrane-bound translational modificator (*mdbA*). In this analysis, the identity threshold percentage was set to 80%.

### 4.5. Phylogenetic Analysis

Whole genome sequences were deposited in BIGSdb-Pasteur for further cgMLST analysis. wgSNPs analysis was performed for all the analyzed strains using CSI Phylogeny 1.4, available on the CGE website [41]. In the next stage of the analysis, five sequences of Belarus strains ST40 were added for a broader analytical context. Because of the fact that the sequences came from different sequencing platforms, only FASTA files were used for comparison.

## Figures and Tables

**Table 1:** Basic data relating to the analyzed strains.

Strain ID	Biotype	Year of Isolation	Patient’s Age	Voivodeship	Source	Other Information
3302/22	gravis	2022	21	Zachodniopomorskie	respiratory tract	
2633/22	mitis	2022	90	Podlaskie	wound	
2102/22	mitis	2022	51	Śląskie	blood	
1781/22	mitis	2022	58	Mazowieckie	unk	
1251/22	mitis	2022	35	Mazowieckie	wound	homeless
648/22	gravis	2022	42	Śląskie	blood	
647/22	gravis	2022	40	Śląskie	blood	
4795/21	gravis	2021	60	Śląskie	unk	
3745/21	gravis	2021	39	Śląskie	blood	
2232/21	gravis	2021	17	Mazowieckie	wound	
1784/21	gravis	2021	41	Śląskie	wound	homeless
1269/21	gravis	2021	55	Warmińsko-mazurskie	blood	
641/21	mitis	2021	39	Mazowieckie	respiratory tract	
576/21	gravis	2021	65	Mazowieckie	blood	
14225/20	gravis	2020	42	Pomorskie	blood	
49390/20	gravis	2020	72	Kujawsko-pomorskie	wound	
38009/20	mitis	2020	62	Kujawsko-pomorskie	eye	homeless *
824/20	mitis	2020	35	Wielkopolskie	blood	
8259/19	gravis	2019	66	Kujawsko-pomorskie	respiratory tract	
5379/19	gravis	2019	59	Pomorskie	blood	homeless
632/19	mitis	2019	66	Mazowieckie	respiratory tract	
230/19	mitis	2019	53	Mazowieckie	wound	homeless *
4674/18	gravis	2018	54	Wielkopolskie	blood	homeless
2736/18	gravis	2018	45	Pomorskie	blood	
5790/18	gravis	2018	59	Mazowieckie	blood	
453/18	gravis	2018	64	Śląskie	wound	homeless *
7072/17	gravis	2017	63	Mazowieckie	unk	homeless
5521/17	gravis	2017	31	Mazowieckie	wound	homeless *
6272/17	mitis	2017	57	Mazowieckie	wound	homeless *
6211/17	gravis	2017	18	Mazowieckie	blood	
54/E	gravis	2015	45	Mazowieckie	wound	homeless
25/E	gravis	2009	unk	Małopolskie	blood	
42/E	gravis	2007	unk	Pomorskie	blood	
17/E	gravis	2007	unk	Kujawsko-pomorskie	wound	
18/E	gravis	2007	unk	Pomorskie	blood	
7/B	mitis	1990s	unk	unk	throat	Diphtheria (tox+)

* According to available data, homelessness is probable, but not confirmed.

**Table 2:** The summary of the main results of the whole genome sequence analyses, including the 7-gene MLST typing, resistance gene profiles, and gene-encoded main *C. diphtheriae* virulence factors.

Strain ID	MLST	Resistance Genes	*tox*	*dtxR*	SpaA-Type Pili (*spaA, spaB, spaC, srtA*)	SpaD-Type Pili (*spaD, spaE, spaF, srtB, srtC*)	SpaH-Type Pili (*spaG, spaH, spaI, srtD, srtE*)	Surface-Anchored Pilus (*sapA, sapD*)	ABC Transporter (*fagA, fagB, fagC, fagD*)	ABC-Type Haem Transporter (*hmuT, hmuU, hmuV*)	Siderophore-Dependent Iron Uptake System (*irp6A, irp6B, irp6C*)	Ciu Iron Uptake and Siderophore Biosynthesis System (*ciuA, ciuB, ciuC, ciuD, ciuE*)	RS 14030	RS 14950	RS 17590	RS 19245	*mdbA*	RS 23695
**Gravis**																		
3302/22	32	-	-	+	+	-	+	+	+	+	+	+	-	+	+	+	+	-
648/22	8	-	-	+	+	+	+	+	+	+	+	+	+	+	+	+	+	+
647/22	8	-	-	+	+	+	+	+	+	+	+	+	+	+	+	+	+	+
4795/21	8	sul1	-	+	+	+	+	+	+	+	+	+	+	+	+	+	+	+
3745/21	8	-	-	+	+	+	+	+	+	+	+	+	+	+	+	+	+	+
2232/21	8	sul1	-	+	+	+	+	+	+	+	+	+	+	+	+	+	+	+
1784/21	8	-	-	+	+	+	+	+	+	+	+	+	+	+	+	+	+	+
641/21	8	sul1	-	+	+	+	+	+	+	+	+	+	+	+	+	+	+	+
576/21	8	-	-	+	+	+	+	+	+	+	+	+	+	+	+	+	+	+
14225/20	8	-	-	+	+	+	+	+	+	+	+	+	+	+	+	+	+	+
49390/20	8	-	-	+	+	+	+	+	+	+	+	+	+	+	+	+	+	+
8259/19	8	sul1	-	+	+	+	+	+	+	+	+	+	+	+	+	+	+	+
5379/19	8	-	-	+	+	+	+	+	+	+	+	+	+	+	+	+	+	+
4674/18	8	-	-	+	+	+	+	+	+	+	+	+	+	+	+	+	+	+
2736/18	8	sul1	-	+	+	+	+	+	+	+	+	+	+	+	+	+	+	+
5790/18	8	sul1	-	+	+	+	+	+	+	+	+	+	+	+	+	+	+	+
453/18	8	sul1	-	+	+	+	+	+	+	+	+	+	+	+	+	+	+	+
7072/17	8	sul1	-	+	+	+	+	+	+	+	+	+	+	+	+	+	+	+
5521/17	8	sul1	-	+	+	+	+	+	+	+	+	+	+	+	+	+	+	+
6211/17	8	-	-	+	+	+	+	+	+	+	+	+	+	+	+	+	+	+
54/E	8	sul1	-	+	+	+	+	+	+	+	+	+	+	+	+	+	+	+
42/E	8	sul1	-	+	+	+	+	+	+	+	+	+	+	+	+	+	+	+
25/E	8	sul1	-	+	+	+	+	+	+	+	+	+	+	+	+	+	+	+
18/E	8	sul1	-	+	+	+	+	+	+	+	+	+	+	+	+	+	+	+
17/E	8	sul1	-	+	+	+	+	+	+	+	+	+	+	+	+	+	+	+
**Mitis**																		
2633/22	819	-	-	+	+/-	-	-	+/-	+	+	+	+	-	+	+	+	+	-
2102/22	40	-	+	+	+/-	-	-	+/-	+	+	+	+	-	+	+	+	+	-
1781/22	439	-	-	+	+/-	+/-	-	+/-	+	+	+	+	+	+	+	+	+	-
1251/22	439	-	-	+	+/-	+/-	-	+/-	+	+	+	+	+	+	+	+	+	-
1269/21	439	-	-	+	+/-	+/-	-	+/-	+	+	+	+	+	+	+	+	+	-
38009/20	439	-	-	+	+/-	+/-	-	+/-	+	+	+	+	+	+	+	+	+	-
824/20	439	-	-	+	+/-	+/-	-	+/-	+	+	+	+	+	+	+	+	+	-
632/19	439	-	-	+	+/-	+/-	-	+/-	+	+	+	+	+	+	+	+	+	-
230/19	439	-	-	+	+/-	+/-	-	+/-	+	+	+	+	+	+	+	+	+	-
6272/17	439	-	-	+	+/-	+/-	-	+/-	+	+	+	+	+	+	+	+	+	-
7/B	44	-	+	+	+/-	-	-	+/-	+	+	+	+	-	+	+	+	+	-

+: positive result; -: negative result; +/-: some but not all genes of the cluster were found.

## Data Availability

All the whole genome sequences were deposited in the GenBank public database as part of BioProject No. PRJNA873913.

## References

[1] Zasada A.A. (2013). Nontoxigenic Highly Pathogenic Clone of *Corynebacterium diphtheriae*, Poland, 2004–2012. Emerg. Infect. Dis..

[2] Zakikhany K., Neal S., Efstratiou A. (2014). Emergence and Molecular Characterisation of Non-Toxigenic *Tox* Gene-Bearing *Corynebacterium diphtheriae* Biovar Mitis in the United Kingdom, 2003–2012. Euro Surveill. Bull. Eur. Sur. Mal. Transm. Eur. Commun. Dis. Bull..

[3] Dangel A., Berger A., Konrad R., Bischoff H., Sing A. (2018). Geographically Diverse Clusters of Nontoxigenic *Corynebacterium diphtheriae* Infection, Germany, 2016–2017. Emerg. Infect. Dis..

[4] Zasada A.A., Baczewska-Rej M., Wardak S. (2010). An Increase in Non-Toxigenic *Corynebacterium diphtheriae* Infections in Poland—Molecular Epidemiology and Antimicrobial Susceptibility of Strains Isolated from Past Outbreaks and Those Currently Circulating in Poland. Int. J. Infect. Dis..

[5] Gubler J., Huber-Schneider C., Gruner E., Altwegg M. (1998). An Outbreak of Nontoxigenic *Corynebacterium diphtheriae* Infection: Single Bacterial Clone Causing Invasive Infection among Swiss Drug Users. Clin. Infect. Dis..

[6] Farfour E., Badell E., Zasada A., Hotzel H., Tomaso H., Guillot S., Guiso N. (2012). Characterization and Comparison of Invasive *Corynebacterium diphtheriae* Isolates from France and Poland. J. Clin. Microbiol..

[7] Fricchione M.J., Deyro H.J., Jensen C.Y., Hoffman J.F., Singh K., Logan L.K. (2014). Non-Toxigenic Penicillin and Cephalosporin-Resistant *Corynebacterium diphtheriae* Endocarditis in a Child: A Case Report and Review of the Literature. J. Pediatr. Infect. Dis. Soc..

[8] Zasada A.A., Zaleska M., Podlasin R.B., Seferyńska I. (2005). The First Case of Septicemia Due to Nontoxigenic *Corynebacterium diphtheriae* in Poland: Case Report. Ann. Clin. Microbiol. Antimicrob..

[9] Walory J., Grzesiowski J., Hryniewicz W. (2001). The Prevalence of Diphtheria Immunity in Healthy Population in Poland. Epidemiol. Infect..

[10] Sangal V., Hoskisson P.A. (2014). Corynephages: Infections of the Infectors. Corynebacterium diphtheriae and Related Toxigenic Species.

[11] Argimón S., Abudahab K., Goater R.J.E., Fedosejev A., Bhai J., Glasner C., Feil E.J., Holden M.T.G., Yeats C.A., Grundmann H. (2016). Microreact: Visualizing and Sharing Data for Genomic Epidemiology and Phylogeography. Microb. Genom..

[12] Czajka U., Wiatrzyk A., Mosiej E., Formińska K., Zasada A.A. (2018). Changes in MLST Profiles and Biotypes of *Corynebacterium diphtheriae* Isolates from the Diphtheria Outbreak Period to the Period of Invasive Infections Caused by Nontoxigenic Strains in Poland (1950–2016). BMC Infect. Dis..

[13] Schaeffer J., Huhulescu S., Stoeger A., Allerberger F., Ruppitsch W. (2021). Assessing the Genetic Diversity of Austrian *Corynebacterium diphtheriae* Clinical Isolates, 2011 to 2019. J. Clin. Microbiol..

[14] Grosse-Kock S., Kolodkina V., Schwalbe E.C., Blom J., Burkovski A., Hoskisson P.A., Brisse S., Smith D., Sutcliffe I.C., Titov L. (2017). Genomic Analysis of Endemic Clones of Toxigenic and Non-Toxigenic *Corynebacterium diphtheriae* in Belarus during and after the Major Epidemic in 1990s. BMC Genom..

[15] Borisova O.I., Mazurova I.K., Chagina I.A., Pimenova A.S., Donskikh E.E., Aleshkin V.A. (2013). Multilocus sequencing of *Corynebacterium diphtheriae* strains isolated in Russia in 2002–2012. Zh. Mikrobiol. Epidemiol. Immunobiol..

[16] Zasada A.A., Rzeczkowska M. (2019). Nontoxigenic *Corynebacterium diphtheriae* Infections, Europe. Emerg. Infect. Dis..

[17] Mandlik A., Swierczynski A., Das A., Ton-That H. (2007). *Corynebacterium diphtheriae* Employs Specific Minor Pilins to Target Human Pharyngeal Epithelial Cells. Mol. Microbiol..

[18] Reardon-Robinson M.E., Ton-That H., Burkovski A. (2014). Assembly and Function of *Corynebacterium diphtheriae* Pili. Corynebacterium diphtheriae and Related Toxigenic Species: Genomics, Pathogenicity and Applications.

[19] Sangal V., Blom J., Sutcliffe I.C., von Hunolstein C., Burkovski A., Hoskisson P.A. (2015). Adherence and Invasive Properties of *Corynebacterium diphtheriae* Strains Correlates with the Predicted Membrane-Associated and Secreted Proteome. BMC Genom..

[20] Peixoto R.S., Antunes C.A., Lourêdo L.S., Viana V.G., Santos C.S.D., Fuentes Ribeiro da Silva J., Hirata R., Hacker E., Mattos-Guaraldi A.L., Burkovski A. (2017). Functional Characterization of the Collagen-Binding Protein DIP2093 and Its Influence on Host-Pathogen Interaction and Arthritogenic Potential of *Corynebacterium diphtheriae*. Microbiol. Read. Engl..

[21] Kolodkina V., Denisevich T., Titov L. (2011). Identification of *Corynebacterium* Diphtheriae Gene Involved in Adherence to Epithelial Cells. Infect. Genet. Evol..

[22] Ott L., Höller M., Gerlach R.G., Hensel M., Rheinlaender J., Schäffer T.E., Burkovski A. (2010). *Corynebacterium diphtheriae* Invasion-Associated Protein (DIP1281) Is Involved in Cell Surface Organization, Adhesion and Internalization in Epithelial Cells. BMC Microbiol..

[23] Sangal V., Hoskisson P.A. (2016). Evolution, Epidemiology and Diversity of *Corynebacterium diphtheriae*: New Perspectives on an Old Foe. Infect. Genet. Evol..

[24] Farfour E., Badell E., Dinu S., Guillot S., Guiso N. (2013). Microbiological Changes and Diversity in Autochthonous Non-Toxigenic *Corynebacterium diphtheriae* Isolated in France. Clin. Microbiol. Infect. Off. Publ. Eur. Soc. Clin. Microbiol. Infect. Dis..

[25] Hoefer A., Pampaka D., Herrera-León S., Peiró S., Varona S., López-Perea N., Masa-Calles J., Herrera-León L. (2021). Molecular and Epidemiological Characterization of Toxigenic and Nontoxigenic *Corynebacterium diphtheriae*, *Corynebacterium* Belfantii, *Corynebacterium* Rouxii, and *Corynebacterium* Ulcerans Isolates Identified in Spain from 2014 to 2019. J. Clin. Microbiol..

[26] Chorlton S.D., Ritchie G., Lawson T., Romney M.G., Lowe C.F. (2020). Whole-Genome Sequencing of *Corynebacterium diphtheriae* Isolates Recovered from an Inner-City Population Demonstrates the Predominance of a Single Molecular Strain. J. Clin. Microbiol..

[27] Timms V.J., Nguyen T., Crighton T., Yuen M., Sintchenko V. (2018). Genome-Wide Comparison of *Corynebacterium diphtheriae* Isolates from Australia Identifies Differences in the Pan-Genomes between Respiratory and Cutaneous Strains. BMC Genom..

[28] Doyle C.J., Mazins A., Graham R.M.A., Fang N.-X., Smith H.V., Jennison A.V. (2017). Sequence Analysis of Toxin Gene-Bearing *Corynebacterium diphtheriae* Strains, Australia. Emerg. Infect. Dis..

[29] Mel’nikov V.G., Kombarova S.I., Borisova O.I., Volozhantsev N.V., Verevkin V.V., Volkovoĭ K.I., Mazurova I.K. (2004). *Corynebacterium diphtheriae* nontoxigenic strain carrying the gene of diphtheria toxin. Zh. Mikrobiol. Epidemiol. Immunobiol..

[30] World Health Organization (2021). WHO Laboratory Manual for the Diagnosis of Diphtheria and Other Related Infections.

[31] Pallen M.J., Hay A.J., Puckey L.H., Efstratiou A. (1994). Polymerase Chain Reaction for Screening Clinical Isolates of Corynebacteria for the Production of Diphtheria Toxin. J. Clin. Pathol..

[32] Hauser D., Popoff M.R., Kiredjian M., Boquet P., Bimet F. (1993). Polymerase Chain Reaction Assay for Diagnosis of Potentially Toxinogenic *Corynebacterium diphtheriae* Strains: Correlation with ADP-Ribosylation Activity Assay. J. Clin. Microbiol..

[33] Bankevich A., Nurk S., Antipov D., Gurevich A.A., Dvorkin M., Kulikov A.S., Lesin V.M., Nikolenko S.I., Pham S., Prjibelski A.D. (2012). SPAdes: A New Genome Assembly Algorithm and Its Applications to Single-Cell Sequencing. J. Comput. Biol..

[34] Lin S.-H., Liao Y.-C. (2013). CISA: Contig Integrator for Sequence Assembly of Bacterial Genomes. PLoS ONE.

[35] Czmil A., Wronski M., Czmil S., Sochacka-Pietal M., Cmil M., Gawor J., Wołkowicz T., Plewczynski D., Strzalka D., Pietal M. (2022). NanoForms: An Integrated Server for Processing, Analysis and Assembly of Raw Sequencing Data of Microbial Genomes, from Oxford Nanopore Technology. PeerJ.

[36] Larsen M.V., Cosentino S., Lukjancenko O., Saputra D., Rasmussen S., Hasman H., Sicheritz-Pontén T., Aarestrup F.M., Ussery D.W., Lund O. (2014). Benchmarking of Methods for Genomic Taxonomy. J. Clin. Microbiol..

[37] Hasman H., Saputra D., Sicheritz-Ponten T., Lund O., Svendsen C.A., Frimodt-Møller N., Aarestrup F.M. (2014). Rapid Whole-Genome Sequencing for Detection and Characterization of Microorganisms Directly from Clinical Samples. J. Clin. Microbiol..

[38] Larsen M.V., Cosentino S., Rasmussen S., Friis C., Hasman H., Marvig R.L., Jelsbak L., Sicheritz-Pontén T., Ussery D.W., Aarestrup F.M. (2012). Multilocus Sequence Typing of Total-Genome-Sequenced Bacteria. J. Clin. Microbiol..

[39] Bortolaia V., Kaas R.S., Ruppe E., Roberts M.C., Schwarz S., Cattoir V., Philippon A., Allesoe R.L., Rebelo A.R., Florensa A.F. (2020). ResFinder 4.0 for Predictions of Phenotypes from Genotypes. J. Antimicrob. Chemother..

[40] Cosentino S., Larsen M.V., Aarestrup F.M., Lund O. (2013). PathogenFinder—Distinguishing Friend from Foe Using Bacterial Whole Genome Sequence Data. PLoS ONE.

[41] Kaas R.S., Leekitcharoenphon P., Aarestrup F.M., Lund O. (2014). Solving the Problem of Comparing Whole Bacterial Genomes across Different Sequencing Platforms. PLoS ONE.

